# Clinical Safety and Performance of Ultra-High Dk Tisilfocon A Orthokeratology Lenses: A Prospective Multicenter Study

**DOI:** 10.3390/life16071143

**Published:** 2026-07-09

**Authors:** Shang-Yen Wu, Jen-Hung Wang, Ti-Yen Cheng, Cheng-Jen Chiu

**Affiliations:** 1 Department of Ophthalmology, Hualien Tzu Chi Hospital, Buddhist Tzu Chi Medical Foundation, Hualien 970, Taiwan; sun90602@gmail.com; 2 Department of Medical Research, Hualien Tzu Chi Hospital, Buddhist Tzu Chi Medical Foundation, Hualien 970, Taiwan; jenhungwang2011@gmail.com; 3 School of Medicine, Tzu Chi University, Hualien 970, Taiwan; 110311151@gms.tcu.edu.tw; 4 Department of Ophthalmology and Visual Science, Tzu Chi University, Hualien 970, Taiwan

**Keywords:** ultra-high Dk Tisilfocon A, orthokeratology (Ortho-K), myopia, prospective multicenter study

## Abstract

This prospective multicenter study evaluated the safety and clinical performance of ultra-high Dk (180 ISO/Fatt) Tisilfocon A orthokeratology (Ortho-K) lenses to treat myopia. We enrolled 67 participants aged 8–49 years with myopia up to 8.00 D. Participants were followed for 36 weeks to assess uncorrected visual acuity (UCVA), axial length (AL), and corneal endothelial cell density (ECD). From the first week, UCVA significantly improved and remained stable through week 36. AL elongation was statistically significant overall, driven primarily by age-appropriate physiological growth in younger participants; adults and individuals with high myopia showed no significant axial elongation. Safety evaluations showed high biocompatibility with no adverse events exceeding Grade 2 corneal staining. ECD remained stable after 36 weeks of lens wear, demonstrating that the ultra-high oxygen permeability of Tisilfocon A supports corneal health and prevents hypoxic stress during overnight wear. Overall, these findings suggest that ultra-high Dk Tisilfocon A Ortho-K lenses are a safe and effective clinical solution for myopia management across diverse patients, providing long-term corneal integrity and stable visual outcomes. ECD remained stable over the 36-week observation period, providing reassuring short- to mid-term evidence that the ultra-high oxygen permeability of Tisilfocon A may help limit hypoxic stress during overnight wear. Overall, these findings suggest that ultra-high Dk Tisilfocon A Ortho-K lenses are well tolerated and clinically effective across diverse patients during this period; however, in the absence of a concurrent control group and given the relatively short follow-up, these results should be regarded as preliminary, and longer-term controlled studies are needed to confirm the durability of axial length control and corneal endothelial safety.

## 1. Introduction

Myopia has become a major global health concern, with particularly high prevalence rates in East Asia. In Taiwan, more than 80% of adolescents are affected [1]. A significant proportion of these individuals will progress to high myopia (≤–6.00 D), which substantially increases lifetime risk of vision-threatening complications like retinal detachment, glaucoma, and myopic maculopathy [1,2,3]. Consequently, early and effective myopia control strategies are needed to mitigate long-term ocular health risks.

Orthokeratology (Ortho-K) has emerged as a nonsurgical intervention for myopia. Using reverse-geometry rigid gas-permeable (RGP) lenses worn overnight, Ortho-K temporarily reshapes the central cornea to correct refractive error while inducing peripheral myopic defocus [4,5,6]. Clinical trials and meta-analyses have demonstrated that Ortho-K can reduce AL elongation by 40–60%, making it a highly effective tool for slowing myopia progression [7,8,9].

However, the safety of overnight lens wear remains a concern. The closed-eye environment during sleep inherently limits oxygen availability, which can lead to corneal hypoxia, edema, and epithelial compromise if the lens material has insufficient oxygen transmissibility (Dk/t). Conventional RGP materials for Ortho-K have Dk values ranging between 85 and 110. Although generally safe for moderate corrections, these materials may still increase the risk of subclinical hypoxia in cases requiring thicker lens designs or in patients with sensitive corneal physiology [1,2,3].

To address these concerns, next-generation RGP materials with progressively higher oxygen permeability have been developed. The CLEAR Orthokeratology report emphasized that while successful orthokeratology outcomes depend primarily on lens fitting, treatment zone centration, corneal morphology, and patient compliance, overnight lens wear inevitably reduces corneal oxygen availability under closed-eye conditions, making oxygen transmissibility an important physiological consideration [10]. Importantly, the use of high oxygen-permeable materials may reduce hypoxic stress, minimize corneal physiological disturbances, and improve corneal tolerance during overnight wear [10]. Supporting this, a clinical study evaluating overnight orthokeratology lenses fabricated from Roflufocon E high-Dk material demonstrated stable refractive correction, acceptable corneal physiological responses, and a favorable safety profile over 36 weeks of follow-up [11]. These findings support the concept that increased oxygen permeability may provide an additional physiological safety margin during overnight orthokeratology treatment, and provide a rationale for investigating materials with even higher oxygen permeability.

Tisilfocon A (Optimum Infinite), with a Dk of 180 (ISO/Fatt), represents a further step forward in oxygen permeability, offering transmissibility substantially beyond that of previously studied materials. Its enhanced oxygen permeability may provide an additional safety margin during overnight wear—particularly in patients requiring greater degrees of corneal reshaping or those with higher baseline myopia necessitating thicker lens designs, as well as across broader age groups. However, whether these material properties translate into clinical benefit remains to be confirmed, as most existing Ortho-K studies have focused on children with low-to-moderate myopia, leaving the safety and efficacy of Tisilfocon A across diverse age groups and a broader myopia spectrum insufficiently established [4,7,12].

The present prospective multicenter study was therefore designed as a confirmatory trial to systematically evaluate the safety and efficacy of Tisilfocon A Ortho-K lenses across a clinically representative population. Specifically, we assessed UCVA, AL stability, corneal ECD, and adverse events in participants spanning a broad age range and myopia spectrum. This intentionally inclusive design was chosen to reflect the diversity of patients encountered in real-world clinical and regulatory settings, and to address the underrepresentation of adults and high myopes in prior orthokeratology research. By providing systematic clinical evidence across these underserved populations, this study aims to establish a robust evidence base for the use of ultra-high Dk Tisilfocon A lenses in contemporary myopia management.

## 2. Materials and Methods

### 2.1. Study Design

This prospective, multicenter, open-label, single-arm clinical study was designed to evaluate the safety and clinical performance of overnight Tisilfocon A Ortho-K lenses. The study was conducted at two tertiary referral centers in Taiwan: the Hualien Tzu Chi General Hospital and Kaohsiung Medical University Chung-Ho Memorial Hospital.

The study protocol adhered to the principles of the Declaration of Helsinki and was approved by the Institutional Review Boards of both participating institutions, including the Institutional Ethical Committee Review Board of Hualien Tzu Chi General Hospital (IRB112-232-A). Written informed consent was obtained from all participants or their legal guardians prior to enrolment.

### 2.2. Participants and Eligibility Criteria

Participants aged 8–49 years with myopia were eligible for inclusion. This broad age range was intentionally selected to reflect real-world orthokeratology practice and includes populations underrepresented in previous studies. Additional inclusion criteria were (i) baseline spherical equivalent (SE) from −0.50 to −8.00 diopters (D); (ii) no prior orthokeratology or refractive surgery; and (iii) willingness and ability to comply with overnight lens wear and follow-up. The exclusion criteria were: (i) active ocular disease or infection; (ii) corneal pathology or irregularity contraindicating Ortho-K lenses; (iii) systemic conditions affecting corneal health; and (iv) history of poor contact lens compliance.

### 2.3. Lens Material and Wear Protocol

All participants were fitted with overnight Ortho-K lenses fabricated from Tisilfocon A (Optimum Infinite^®^, Contamac Ltd., Saffron Walden, UK), an ultra-high oxygen permeability material with a reported Dk value of 180 (ISO/Fatt). Lens design followed standard reverse-geometry principles, with individualized parameter adjustments based on corneal topography and refractive error. Lens fitting was assessed using fluorescein staining to evaluate lens centration, with a thin annular layer of tears trapped in the reverse curve forming a bull’s-eye configuration. Good centration with appropriate tightness was defined as mild upward movement of the Ortho-K lens after blinking. The same practitioner (CJC) fitted all Ortho-K lenses using standardized criteria [13].

Participants were instructed to wear the lenses for at least 8 h nightly. Standardized lens care instructions were provided, including daily cleaning with a povidone iodine-based rigid lens disinfecting solution or a multipurpose solution with lens rubbing, biweekly protein removal, and replacement of the lens case and holder every 3 months.

### 2.4. Follow-Up Schedule and Assessments

Follow-up visits were scheduled at weeks 1, 4, 12, 24, and 36. The 36-week follow-up duration was selected to be consistent with previously published orthokeratology safety studies evaluating highly oxygen-permeable lens materials [11,14]. Although the primary follow-up period was 36 weeks, most participants were additionally invited to return at week 48 for an exploratory axial length assessment, given that myopia-control studies commonly evaluate AL changes over approximately one year. At each follow-up visit, the following assessments were performed: UCVA and logMAR scale, AL measurement using optical biometry (measured in triplicate and averaged), manifest refraction, keratometry and corneal topography, slit lamp examination (graded using the CCLRU scale by the same physician throughout the study), and adverse event reporting. Ocular surface findings, including corneal staining, were graded by the same physician throughout the study using the Cornea and Contact Lens Research Unit (CCLRU) grading scale [15], a standardized 0–4 scale based on photographic reference standards (0 = none; 1 = trace/mild, scattered superficial punctate staining; 2 = mild-to-moderate punctate or focal staining without epithelial compromise; 3 = moderate, confluent staining with possible mild epithelial involvement; 4 = severe staining with frank epithelial defect, stromal involvement, or infiltrate). ECD was measured at baseline and at study completion using the Konan Noncon Robo SP8000 (Konan Medical, Hyogo, Japan), a specular microscope with established measurement reliability [16]. Each eye was measured three times per visit, and the mean value was recorded for analysis. To ensure measurement consistency, all follow-up examinations were completed before 11:00 a.m., within 2 h of lens removal.

### 2.5. Statistical Analysis

Statistical analysis was performed using SPSS version 21.0. Data are reported as frequencies, proportions, or means ± standard deviations (SD), as appropriate. The Kolmogorov–Smirnov test was used to assess the normality of the data. Associations between categorical variables were assessed using the chi-square test or Fisher’s exact test. The independent *t*-test or Wilcoxon rank-sum test was used to compare the means of continuous variables between two groups. Generalized estimating equations (GEE) were used to evaluate overall time trends, providing a single estimate of temporal change without requiring multiple pairwise comparisons. As both eyes were analyzed, left and right eyes were assessed separately, thereby avoiding potential bias introduced by inter-eye correlation. To reduce the risk of an inflated overall type I error arising from multiple statistical tests, a more stringent two-sided significance threshold of *p* < 0.01 was used.

Sample size was estimated using an online calculator (http://powerandsamplesize.com/Calculators/Test-1-Proportion/1-Sample-1-Sided) accessed on 22 March 2026, based on the proportion of participants expected to meet predefined safety and efficacy criteria, consistent with the confirmatory trial framework of this study. The study hypothesis was H0: *p* = P_0_ vs. H1: *p* > P_0_. Under the assumptions of *p* = 0.95, P_0_ = 0.85, α = 0.025, and β = 0.20, the required sample size was approximately 60 participants in total (from the two hospitals). As we anticipated a dropout rate of 10–15%, the recruitment target was approximately 70 participants, with 35 recruited at each hospital.

## 3. Results

### 3.1. Participant Characteristics

We recruited a total of 67 participants (134 eyes) who completed the 36 weeks follow-up. Table 1 provides a summary of the participants’ demographic characteristics. The mean age was 18.19 ± 10.31 years and 59.7% of participants were female. Age and refractive error distributions were well balanced across predefined subgroups.

Baseline SE ranged from mild to high myopia, with a substantial proportion of participants exceeding the threshold of −5.00 D for high myopia.

### 3.2. Overall Refractive and Visual Outcomes

Table 2 presents the observed time trends of the covariates across the 36-week follow-up period, with an exploratory axial length assessment at week 48. Rapid improvement in UCVA was observed by week 1, accompanied by significant reductions in residual refractive error (RRE) and central corneal curvature. These changes stabilized by week 4 and were maintained through week 36.

- Corneal reshaping: Mean flat keratometry (Flat K) decreased significantly from baseline by week 1 for both the right (from 42.91 ± 1.35 D to 40.73 ± 1.65 D) and left eyes (from 42.96 ± 1.36 D to 40.91 ± 1.68 D) and remained stable thereafter (*p* < 0.001 for both eyes).
- Visual acuity: Mean logMAR UCVA improved dramatically from baseline (right eye: 1.93 ± 0.91; left eye: 1.92 ± 0.86) to week 1 (right eye: 0.24 ± 0.36; left eye: 0.28 ± 0.45; *p* < 0.001). By week 36, logMAR UCVA had further stabilized to 0.11 ± 0.13 (right eye) and 0.12 ± 0.15 (left eye).
- Residual refractive error (RRE): Mean RRE decreased significantly from baseline at all measured time points (*p* < 0.001). At week 36, RRE had stabilized at −0.47 ± 0.70 D in the right eye and −0.47 ± 0.65 D in the left eye.

### 3.3. Analysis Stratified by Age

Similar temporal patterns of corneal reshaping and visual improvement were observed across all age groups (Table S1). Specifically, children aged 8–12 years showed rapid visual improvement and stable corneal flattening, with no increase in adverse events. The stabilization timelines were similar for adolescents (13–18 years). Adults (≥19 years) showed slightly smaller magnitudes of corneal flattening, but achieved comparable UCVA outcomes. The lack of a significant age-by-time interaction suggests age-independent efficacy of treatment.

### 3.4. Analysis Stratified by Myopia Severity

Participants with high myopia (defined as >−5.00 D) exhibited slightly slower initial refractive neutralization. However, by week 12, UCVA and refractive outcomes were comparable to those of participants with lower myopia (Table S2). Safety outcomes did not differ by refractive severity, supporting the feasibility of Tisilfocon A Ortho-K lenses for high myopia.

### 3.5. AL Outcomes

AL showed a statistically significant overall time trend throughout the 36-week follow-up period (*p* < 0.001, Table 2). Stratified by age group (Table S1), significant AL elongation was observed in children aged 8–12 years and adolescents aged 13–18 years, consistent with expected physiological ocular growth in developing eyes. In contrast, adults aged ≥19 years showed no significant AL trend (right eye: *p* = 0.184; left eye: *p* = 0.531). Among participants aged 8–12 years, the magnitude of AL elongation was comparable to or lower than that reported in similarly aged children undergoing orthokeratology monotherapy in previous studies [17]. Stratified by baseline SE (Table S2), participants with mild (≤−3.0 D) and moderate myopia (−3.0 D to −5.0 D) showed modest but statistically significant AL increases, whereas those with high myopia (>−5.0 D) showed no significant change (right eye: *p* = 0.952; left eye: *p* = 0.286).

### 3.6. Safety Outcomes

Across 670 eye examinations, safety assessments confirmed a favorable profile for the Tisilfocon A material. Adverse events and complaints were infrequent and mild. The most common participant-reported complaints were transient foreign body sensation (eight cases, ~1.2% of participant × visit), poor distant vision (10 cases, ~1.5% of participant × visit), and tearing (two cases, ~0.3% of participant · visit), all primarily confined to the first week of adaptation (Table S3). The slit lamp findings revealed one case of Grade 1 corneal staining in the right eye and two cases in the left eye, likely related to early adaptation. Other minor findings included allergic conjunctivitis (seven total cases). Crucially, no cases of Grade 2 or 3 corneal staining, corneal edema, keratitis, corneal ulcers, scarring, or neovascularization were observed (Table S4).

#### ECD Results

Endothelial cell counts remained stable from baseline to completion of treatment (Table 3). Besides, over the 36 weeks follow-up period, no significant differences were observed in Hexagonality or Coefficient of Variation (all *p* > 0.05). Preservation of surface integrity was also clinically confirmed upon retrieval of the lenses at 36 weeks.

## 4. Discussion

To our knowledge, this is among the first clinical studies to comprehensively evaluate the safety and clinical performance of ultra-high Dk Tisilfocon A Ortho-K lenses across a broad age range and extended myopia spectrum. Unlike previous studies that primarily focused on pediatric populations with low-to-moderate myopia, our sample included adults and individuals with higher refractive errors, providing a more representative reflection of real-world clinical practice. Over the 36-week follow-up period, these lenses were well tolerated with infrequent and mild adverse events, stable ECD, and preserved lens surface integrity [18]. Importantly, these outcomes were consistent across age and refractive severity subgroups, supporting the generalizability of the findings [18,19].

### 4.1. Safety Profile of Ultra-High Dk Material

The primary physiological concern associated with overnight Ortho-K lenses is corneal hypoxia due to reduced oxygen availability when eyes are closed. Previous generations of RGP materials, which typically have Dk values in the range of 85–110, are considered clinically acceptable [20]. However, subclinical hypoxic stress is still possible—particularly in patients requiring greater corneal reshaping or extended wearing times [20,21]. As the ultra-high Dk material (Dk = 180) is designed to provide a substantial increase in oxygen permeability, it may provide an additional safety margin.

The safety outcomes in this study are consistent with a favorable physiological profile of Tisilfocon A. The absence of clinically significant corneal complications—including Grade ≥ 2 staining, corneal edema, or neovascularization—suggests that corneal epithelial integrity was well maintained. ECD was stable over the 36-week period, suggesting preservation of corneal endothelial function. Endothelial cell density has been widely used as a surrogate marker for chronic hypoxic stress during contact lens wear, given the known susceptibility of corneal endothelium to oxygen deprivation under closed-eye conditions [10,21]. Because endothelial cells are non-regenerative, this finding may serve as an important indicator of long-term safety. Notably, given the inherently slow dynamics of endothelial changes, the absence of significant ECD loss over the 36-week follow-up period is consistent with expectations for a well-tolerated overnight lens material and supports its corneal safety profile. ECD was stable over the 36-week period, which is a reassuring early signal regarding corneal endothelial function. Endothelial cell density has been widely used as a surrogate marker for chronic hypoxic stress during contact lens wear, given the known susceptibility of corneal endothelium to oxygen deprivation under closed-eye conditions [10,22]. However, because endothelial cells are non-regenerative and typically undergo slow, cumulative loss in response to chronic hypoxic stress, this same time course is also an important limitation of our findings: subclinical endothelial stress, if present, may not become statistically or clinically detectable within a 36-week window, particularly in a single-arm design without a concurrent lower-Dk comparator against which to benchmark the expected rate of physiological cell loss. Definitive characterization of the long-term endothelial safety of this ultra-high Dk material will therefore require follow-up extending over several years, ideally incorporating a control arm. Moreover, there were no clinically evident concerns regarding surface durability and protein deposition associated with the silicone-containing high-Dk materials. The lens surfaces appeared intact upon clinical inspection at study completion, and no increase in lens-related complications was reported. These findings suggest that Tisilfocon A may provide acceptable mechanical and surface stability under routine clinical use [23,24].

These safety findings are broadly consistent with those reported in previous clinical studies evaluating high oxygen-permeable orthokeratology materials. Cheng et al. reported a favorable short-term safety profile with Boston XO2 lenses (Dk = 141) over 24 weeks, including stable corneal physiology and no serious adverse events [14]. Similarly, Huang et al. observed acceptable corneal physiological responses and preserved ECD over 36 weeks with Roflufocon E lenses [11]. The present findings with Tisilfocon A (Dk = 180) are consistent with these earlier reports, suggesting that ultra-high Dk materials are well tolerated under overnight orthokeratology conditions.

Mechanistically, higher oxygen transmissibility (Dk/t) is expected to reduce epithelial edema and maintain normal corneal metabolism under hypoxic stress [19,20]. Although increased Dk may reduce hypoxia-related risk, it is important to note that the relationship between material oxygen permeability and long-term clinical outcomes is multifactorial and influenced by lens design, fit, tear exchange, and patient compliance [21,24]. The present findings should therefore be interpreted as supportive rather than definitive evidence of a physiological advantage.

### 4.2. Efficacy and Broad Applicability

In addition to its favorable safety profile, Tisilfocon A Ortho-K lenses demonstrated effective and stable refractive outcomes in the present study. The observed rapid improvement in UCVA and corneal flattening is consistent with the expected response to reverse-geometry lens design, indicating a sustained treatment effect [25]. Importantly, treatment responses were consistent across age groups. Although adults exhibited slightly smaller magnitudes of corneal reshaping, the final visual outcomes were comparable to those observed in children and adolescents. These findings suggest that orthokeratology with ultra-high Dk material may remain effective even in populations with potentially reduced corneal plasticity. Furthermore, participants with higher baseline myopia (up to −8.00 D) achieved comparable refractive outcomes after the initial adaptation period. This is clinically relevant because high myopia has traditionally been considered a relative limitation for orthokeratology, primarily due to the difficulty of achieving sufficient corneal reshaping and maintaining an adequately centered treatment zone across a broader optical zone [25,26]. The present findings suggest that the use of ultra-high Dk material may help mitigate these concerns, potentially enabling effective treatment in a broader range of patients. The refractive and visual outcomes observed in the present study are also comparable to those reported by Cheng et al. and Huang et al., in which stable refractive correction and satisfactory UCVA were achieved with high-Dk orthokeratology lenses over similar follow-up periods [11,14]. Taken together, these findings support the applicability of Tisilfocon A lenses across a wide clinical spectrum, including populations underrepresented in previous research [26,27,28].

### 4.3. Control of AL

AL showed a statistically significant overall time trend over the 36-week observation period (*p* < 0.001), primarily driven by younger age groups undergoing expected physiological ocular growth [16]. Stratified analyses revealed that adults aged ≥19 years and participants with high myopia (>−5.0 D) showed no significant AL elongation, suggesting that the observed overall trend reflects developmental growth rather than pathological progression. This pattern is consistent with early-phase responses reported in previous orthokeratology studies where suppression of axial elongation was most pronounced during the initial treatment period [29,30,31].

Orthokeratology has been shown to reduce axial elongation by approximately 40–60% relative to single-vision correction, with previously reported annual differences of approximately 0.20–0.30 mm [32,33,34,35]. The stability observed in the present study is comparable to these early treatment-phase findings. However, as our study lacked a control group, direct comparisons with other treatment modalities or lens materials cannot be made. The stability observed in the present study is comparable to these early treatment-phase findings. However, as our study lacked a concurrent control group, direct comparisons with other treatment modalities or lens materials cannot be made, and the contribution of this lens material specifically—as opposed to natural variation in growth rate or age-related deceleration of axial elongation—cannot be isolated. Furthermore, because axial elongation is a cumulative process conventionally characterized over multi-year intervals in myopia-control trials, our 36-week (and limited 48-week exploratory) observation window is insufficient to determine whether the absence of significant AL elongation in older or highly myopic participants reflects a genuine treatment effect, an age- or refraction-related ceiling effect on an already slow growth rate, or simply an observation period too short to detect a difference. Longer prospective follow-up incorporating a concurrent control group will be required to confirm both the magnitude and durability of AL control attributable to this material.

Importantly, the use of ultra-high Dk material did not appear to compromise AL control [36,37]. It should be noted that oxygen transmissibility itself is not considered a direct biological mechanism underlying orthokeratology-related myopia control, which is primarily attributed to corneal reshaping and alteration of peripheral retinal defocus. Nevertheless, improved oxygen permeability may enhance physiological tolerance and overnight wearing comfort, potentially supporting better treatment adherence and long-term compliance—factors that may indirectly contribute to sustained myopia control in clinical practice [38]. Thus, while the findings support clinical feasibility [22,27,39], they do not establish superiority. The inclusion of younger children and individuals with higher myopia—populations frequently underrepresented in orthokeratology research—and the observation of clinically meaningful outcomes within these groups strengthen the external validity of this study [19,23,24]. However, it should be noted that the stratified subgroup analyses were limited by relatively modest sample sizes within each age group, which may have reduced statistical power to detect subtle differences in AL progression dynamics across pediatric and adult populations. This remains an important limitation to be addressed in future studies with larger sample sizes.

### 4.4. Clinical Implications of Ultra-High Dk Materials in Real-World Orthokeratology Practice

Beyond refractive correction and AL control, material properties play a critical role in the real-world success of orthokeratology [39,40,41,42]. Although patient-reported outcomes were not formally assessed in this study, indirect observations regarding symptom frequency and clinical tolerability are discussed below in the context of material properties. Factors such as wearing comfort, physiological tolerance, and ease of maintenance directly influence patient adherence and long-term treatment outcomes. Overnight lens wear inherently places the cornea in a relatively hypoxic environment [43,44]. Although the relationship between Dk/t and corneal oxygen availability under closed-eye conditions may become asymptotic beyond certain thresholds, materials with substantially higher Dk values—such as Tisilfocon A (Dk = 180)—may still provide a meaningful physiological margin, particularly in patients requiring thicker lens designs or extended wear durations [45,46].

Reports of subjective symptoms were infrequent and largely confined to the early adaptation phase in the present study. There was no clear pattern linking these symptoms to certain age or refractive severity groups, which suggests that ultra-high Dk materials may support a more uniform wearing experience across diverse patients [18,47,48]. One possible explanation is that enhanced oxygen permeability may improve corneal physiological stability during overnight wear.

These observations are particularly relevant in the context of pediatric myopia management, as long-term compliance is essential for treatment success [49]. Even modest gains in comfort and tolerance may translate into meaningful effects on adherence over time. While we did not formally assess patient-reported outcomes, the low frequency of discomfort-related complaints supports the feasibility of Tisilfocon A lenses in routine clinical practice.

### 4.5. Study Limitations and Future Directions

First, the single-arm design without a control group is consistent with the confirmatory trial framework of this study, but limits the ability to draw direct material comparisons. Second, although the follow-up duration is comparable to similarly designed orthokeratology trials, it remains insufficient for fully characterizing long-term AL progression, particularly in younger children. Third, subgroup sample sizes were relatively modest, which may limit statistical power for detecting subtle intergroup differences. Fourth, while lens surface integrity was clinically assessed, laboratory-based analyses would be needed to quantify protein deposition and long-term material degradation. Last, as this was an open-label single-arm study, formal masking of the examiner to the study hypothesis was not feasible, which may have introduced assessment bias in subjective grading outcomes.

Future studies should incorporate randomized controlled designs, direct comparisons of materials, and longer follow-up durations. In particular, studies in pediatric populations and examining long-term AL control will be critical for determining whether ultra-high Dk materials provide clinically meaningful advantages over existing options for myopia control.

First, the absence of a concurrent control group limits this study’s ability to attribute the observed AL stability and safety findings specifically to Tisilfocon A, rather than to natural variation, age-related growth deceleration, or regression to the mean; comparative claims relative to other materials or treatment modalities should therefore be regarded as hypothesis-generating rather than confirmatory, pending validation in a controlled trial.

Second, the 36-week observation period (with a limited 48-week exploratory AL assessment) is likely too short to fully characterize either the long-term AL-control effect—conventionally assessed over 1–3 years—or the long-term safety profile of this material. Endothelial cell loss from chronic hypoxic stress, and material degradation such as surface wettability or protein deposition both follow slow, cumulative time courses that may remain subclinical well beyond 36 weeks. The favorable ECD and surface-integrity findings reported here should therefore be regarded as reassuring short- to mid-term observations rather than definitive evidence of long-term safety; longer-term studies incorporating a control arm are needed to confirm these findings.

Third, the relatively modest subgroup sample sizes may have limited statistical power to detect subtle intergroup differences, and the lack of examiner masking inherent to this open-label design may have introduced some assessment bias in subjective grading outcomes.

Nevertheless, the primary safety and efficacy hypotheses of this confirmatory trial were met, with no safety signal observed across an expanded population including adults and individuals with high myopia—groups previously underrepresented in orthokeratology research. These findings support the use of Tisilfocon A in this broader population while underscoring the need for longer-term, controlled studies to confirm its long-term advantages.

Future studies should incorporate randomized controlled designs, direct material comparisons, and longer follow-up durations, particularly to determine whether ultra-high Dk materials provide clinically meaningful advantages over existing options for myopia control.

## 5. Conclusions

Despite the limitations of this study, Tisilfocon A Ortho-K lenses demonstrated consistent refractive efficacy, AL stability, and a favorable safety profile across a broad age range and spectrum of myopia severity. These findings support the clinical feasibility of ultra-high Dk materials in contemporary orthokeratology practice and underscore the need for long-term comparative studies. Despite the limitations of this study—most notably the absence of a concurrent control group and the relatively short, 36-week observation period—Tisilfocon A Ortho-K lenses demonstrated consistent refractive efficacy, short- to mid-term AL stability, and a favorable safety profile across a broad age range and spectrum of myopia severity. These findings support the clinical feasibility of ultra-high Dk materials in contemporary orthokeratology practice but should not be interpreted as confirmatory evidence of long-term axial elongation control or definitive endothelial and material safety. Longer-term, controlled comparative studies—incorporating extended follow-up of endothelial cell density, AL trajectories, and material durability—are needed to substantiate these preliminary findings.

## Figures and Tables

**Table 1:** Demographics (*n* = 67).

Variable	Male	Female	Total	*p*-Value
Numbers	27	40	67	
Age	14.56 ± 5.72	20.65 ± 11.95	18.19 ± 10.31	0.016
Age Group (%)				0.120
8–12 y/o	10 (37.0%)	14 (35.0%)	24 (35.8%)	
13–18 y/o	12 (44.4%)	10 (25.0%)	22 (32.8%)	
≥19 y/o	5 (18.5%)	16 (40.0%)	21 (31.3%)	
Enrolment Site (%)				0.052
Tzu-Chi	18 (66.7%)	17 (42.5%)	35 (52.2%)	
KMUH	9 (33.3%)	23 (57.5%)	32 (47.8%)	
SE (Right Eye) Groups (%)				0.221
≤−3.0 D	13 (48.1%)	13 (32.5%)	26 (38.8%)	
−3.0 D to −5.0 D	9 (33.3%)	12 (30.0%)	21 (31.3%)	
>−5.0 D	5 (18.5%)	15 (37.5%)	20 (29.9%)	
SE (Left Eye) Groups (%)				0.578
≤−3.0 D	11 (40.7%)	15 (37.5%)	26 (38.8%)	
−3.0 D to −5.0 D	9 (33.3%)	10 (25.0%)	19 (28.4%)	
>−5.0 D	7 (25.9%)	15 (37.5%)	22 (32.8%)	
Right Eye				
Baseline AL	25.37 ± 1.09	24.92 ± 1.10	25.15 ± 1.10	0.238
Baseline FK	42.22 ± 1.16	43.38 ± 1.27	42.91 ± 1.35	<0.001 *
Baseline UCVA	0.29 ± 0.24	0.17 ± 0.16	0.22 ± 0.20	0.021
Baseline logMAR	1.64 ± 0.96	2.12 ± 0.84	1.93 ± 0.91	0.034
Baseline Sphere	−3.29 ± 1.95	−3.94 ± 1.97	−3.68 ± 1.98	0.188
Baseline Cylinder	−0.79 ± 0.76	−0.83 ± 0.76	−0.81 ± 0.76	0.817
Baseline SE	−3.68 ± 2.06	−4.35 ± 2.13	−4.08 ± 2.11	0.204
Baseline Endothelial Cell Counts	3070.19 ± 233.96	3081.58 ± 245.78	3076.99 ± 239.35	0.850
Left Eye				
Baseline AL	25.45 ± 1.10	24.77 ± 1.06	25.12 ± 1.12	0.071
Baseline FK	42.28 ± 1.24	43.42 ± 1.25	42.96 ± 1.36	<0.001 *
Baseline UCVA	0.25 ± 0.18	0.18 ± 0.18	0.21 ± 0.19	0.102
Baseline logMAR	1.69 ± 0.88	2.07 ± 0.82	1.92 ± 0.86	0.071
Baseline Sphere	−3.31 ± 2.16	−3.87 ± 1.93	−3.64 ± 2.03	0.269
Baseline Cylinder	−0.97 ± 0.81	−0.94 ± 0.83	−0.96 ± 0.81	0.890
Baseline SE	−3.79 ± 2.2	−4.34 ± 2.08	−4.12 ± 2.13	0.305
Baseline Endothelial Cell Counts	3107.44 ± 246.99	3047.58 ± 288.31	3071.7 ± 272.08	0.381

Data are presented as *n* (%) or mean ± standard deviation. * *p*-value < 0.01 was considered statistically significant after test.

**Table 2:** Time trends of covariates during follow-up period (*n* = 67).

Variable	Baseline	1 W	4 W	12 W	24 W	36 W	48 W	*p* for Trend ^a^
Right Eye								
Flat K	42.91 ± 1.35	40.73 ± 1.65	40.39 ± 1.77	40.32 ± 1.85	40.24 ± 1.74	40.37 ± 1.72	NA	<0.001 *
logMAR	1.93 ± 0.91	0.24 ± 0.36	0.16 ± 0.32	0.12 ± 0.16	0.12 ± 0.17	0.11 ± 0.13	NA	<0.001 *
Residual Refractive Error	−4.08 ± 2.11	NA	−0.51 ± 1.03	−0.37 ± 0.66	−0.32 ± 0.57	−0.47 ± 0.70	NA	<0.001 *
Axial Length	25.15 ± 1.10	NA	NA	25.16 ± 1.08	25.18 ± 1.06	25.20 ± 1.05	25.25 ± 1.06	<0.001 *
Left Eye								
Flat K	42.96 ± 1.36	40.91 ± 1.68	40.49 ± 2.04	40.37 ± 1.83	40.42 ± 1.78	40.50 ± 1.69	NA	<0.001 *
logMAR	1.92 ± 0.86	0.28 ± 0.45	0.17 ± 0.21	0.13 ± 0.16	0.14 ± 0.17	0.12 ± 0.15	NA	<0.001 *
Residual Refractive Error	−4.12 ± 2.13	NA	−0.57 ± 0.86	−0.34 ± 0.62	−0.43 ± 0.72	−0.47 ± 0.65	NA	<0.001 *
Axial Length	25.12 ± 1.12	NA	NA	25.12 ± 1.10	25.14 ± 1.09	25.17 ± 1.08	25.22 ± 1.07	<0.001 *

Data are presented as mean ± standard deviation. * *p*-value < 0.01 was considered statistically significant after test. ^a^ *p*-value for time in the generalized estimating equations model after adjustment for covariates, including age, sex, and time.

**Table 3:** Comparison of change of endothelial cell counts, hexagonality, and CV before and after trial (*n* = 67).

Item	Side	*N*	Before	After	Diff. (After-Before)	*p*-Value
Endothelial cell counts	Right	67	3076.99 ± 239.35	3093.54 ± 252.20	16.55 ± 149.32	0.368
	Left	67	3071.70 ± 272.08	3106.48 ± 260.24	34.78 ± 164.87	0.089
Hexagonality (HEX)	Right	67	62.94 ± 5.72	63.20 ± 7.50	0.26 ± 6.77	0.824
	Left	67	63.46 ± 6.79	64.20 ± 5.31	0.74 ± 6.94	0.531
Coefficient of variation (CV)	Right	67	37.49 ± 7.31	36.57 ± 8.47	−0.91 ± 7.09	0.451
	Left	67	36.89 ± 8.13	35.91 ± 4.85	−0.97 ± 5.82	0.331

Data are presented as mean ± standard deviation. *p*-value < 0.01 was considered statistically significant after test.

## Data Availability

The data presented in this study are available on request from the corresponding author. The data are not publicly available due to patient confidentiality.

## Supplementary Materials

The following supporting information can be downloaded at https://www.mdpi.com/article/10.3390/life16071143/s1, Table S1: Time trends of covariates during follow-up period stratified by age groups (*n* = 67); Table S2: Time trends of covariates during follow-up period stratified by SE groups (*n* = 67); Table S3. Self-reported symptoms, problems, and complains during follow-up period; Table S4. Slit lamp examination results during follow-up periods.
